# ^1^H NMR plasma metabolomic profiling of ovarian quiescence in energy balanced postpartum dairy cows

**DOI:** 10.1080/01652176.2018.1473660

**Published:** 2018-06-27

**Authors:** Jiang Zhang, Gang Wang, Chang Zhao, Yunlong Bai, Shi Shu, Ziling Fan, Cheng Xia

**Affiliations:** Department of Animal Science and Veterinary Medicine, Heilongjiang Bayi Agricultural University, Daqing, China

**Keywords:** Cow, bovine, anestrus, NMR, metabolomics

## Abstract

**Background:** As the milk production of dairy cows increases, the reproductive capacity gradually declines. Ovarian quiescence has become one of the concerns of the dairy industry.

**Objective:** To explore the different plasma metabolite levels between estrus and anestrus in energy balanced (EB) dairy cows.

**Methods:** Ten estrous and 10 anestrus EB Holstein cows in early lactation were selected for the study. ^1^H nuclear magnetic resonance technology was used to detect plasma metabolites and screen different plasma metabolites between anestrous and estrous cows at 60–90 days postpartum using multivariate statistical analysis.

**Results:** Within an elective waiting period of 60−90 days postpartum mean plasma concentration of total estrogens was significantly higher in estrus cows as compared to anestrus cows (71.2 ± 26.0 and 42.4 ± 16.7 pg/mL, respectively). Seven plasma metabolites (isoleucine, leucine, valine, alanine, arginine, choline and phosphatecholine) demonstrated significant decreases in estrous dairy cows relative to anestrous subjects. The main pathway was leucine, isoleucine and valine biosynthesis.

**Conclusion:** Anestrus in dairy cows is accompanied by alterations in amino acid, glucose and lipid metabolism based on ^1^H NMR analysis.

## 1. Introduction

In recent years, with the increase of average milk yield in the global dairy industry, anestrus in high-producing dairy cows is becoming increasingly problematic (Jena et al. 2016), causing low reproduction rates, extended periods of infertility and calving intervals, with serious consequences to dairy farm economics. Ovarian quiescence has been accepted as the most common cause of anestrus in high-producing cows. Xu C-C et al. (2016) reported a relationship between ovarian quiescence and negative energy balanced (NEB) in a ^1^H NMR metabolomics study. Walsh et al. (2011) also pointed out that NEB is the main cause of ovarian quiescence in dairy cows. Early lactation cows have the risk of ketosis. Type II ketosis is associated with intense fat mobilization, and type I ketosis is mainly due to a lack of intake of gluconeogenic precursors. This study selected energy balanced (EB) dairy cows to reduce the impact of ketosis and negative energy balance on the results.

NMR is a proven metabolomics technology and has the advantages of simple sample pretreatment, no bias to the sample, and no damage caused during analysis. In this study, ^1^H NMR metabolomics combined with multivariate statistical analysis was used to compare the metabolic profiles of EB cows with ovarian quiescence to those with normal estrus during early lactation. The relevance of the different metabolite levels detected in ovarian quiescence compared to normal ovarian activity and for future prevention and/or treatment of postpartum anestrus is discussed.

## 2. Materials and methods

### 2.1. Animals

The study was administrated strictly according to the Guide for the Care and Use of Laboratory Animals of the National Institutes of Health. All experiments on animals were carried out according to the standards approved by the Animal Welfare and Research Ethics Committee at Heilongjiang Bayi Agricultural University.

There were 1045 Holstein dairy cows in the herd with an average calving interval of 393 days. Average milk yield comprised 7000 L per cow per year with 3.8% protein and 4.7% fat. The total mixed ration of tested dairy cows complied with the Chinese Feeding Standard of Dairy Cows and consisted of 1 kg Oat Hay, 2.8 kg alfalfa, 10.4 kg corn silage, 3 kg wet corn, 2.7 kg cottonseed, 8.7 kg premixes and water ad libitum. The nutrient level was dry matter: 55.6%, crude protein (CP): 16%, fat: 5.60%, Ca: 180 g, P: 116 g, neutral detergent fiber: 39.10%, acid detergent fiber: 20.30% and net energy for lactation: 1.75 MJ/kg. Tested cows were milked three times a day.

### 2.2. Sample collection

EB cows, 14–21 days postpartum (Holstein; parity 3.25 ± 0.50; 650 ± 54 kg body weight; average milk yield of 33.2 ± 1.7 kg/day; age 3.33 ± 1.51 years) were selected based on their plasma concentrations of β-hydroxybutyric acid (BHBA), non-esterified fatty acids (NEFA) and glucose (<1.2, <0.5 and >2.8 mmol/L, respectively).

The estrous or anestrous state of selected cows was determined over an elective waiting period of 60−90 days postpartum from assessment of estrus behavior, rectal examination and B ultrasound examination every three days. Estrous cows were defined as those with obvious symptoms of estrus, no uterine or ovarian abnormalities and the presence of a mature follicle. If these signs continued for seven days, the cows were included in the study. Anestrous cows associated with abnormal ovarian follicle dynamics were defined as those failing to show (metestrous) bleeding or demonstrating abnormal bleeding and no significant changes over a seven-day observation period (Rajamahendran et al. 1994). Blood samples (10 mL) were collected from estrous and anestrous cows at two times namely 14–21 days and 60–90 days postpartum (10 biological replicates each) via coccygeal venipuncture into vials containing heparin sodium and centrifuged at 3000 × *g* for 10 min at room temperature before the supernatants (plasma) were stored at −80 °C until analysis. At both time points plain tubes (for BHBA and NEFA's) and tubes containing NaF (for glucose) were collected. In addition, at the second time point vials containing heparin sodium were collected for ^1^H NMR analysis as well as progesterone and total estrogens.

### 2.3. Detection of plasma hormones and biochemical parameters

Plasma biochemical parameters and hormones were detected using an automatic biochemical analyzer (HITACHI 7170, Tokyo, Japan) and multimode reader (Tecan Infinite® 200 Pro, M200 PRO, Salzburg, Austria). Commercial kits were used to assay 3-hydroxybutyrate, NEFA (Beijing Strong Biotechnologies Inc., Beijing, China) and glucose (BioSino Bio-Technology & Science Inc., Shanghai, China). Total estrogens and progesterone were assayed by ELISA kits (Nanjing SenBeiJia Biological Technology Co., Ltd., Nanjing, China). The resulting data were analyzed by SPSS 19.0 (SPSS, IBM, Armonk, NY, USA) using an unpaired *t*-test.

### 2.4. ^1^H NMR analysis of blood plasma

Heparin plasma samples taken at 60–90 days postpartum were used for 1H NMR analysis and adjusted to 66.53% (v/v) D_2_O phosphate buffer (0.2 M Na_2_HPO, 0.2 M NaH_2_PO_4_ and 0.05% (v/v) TSP), centrifuged at 12 000 × *g* for 20 min at 4 °C and the supernatants probed with a Bruker 600 MHz ultra-low temperature probe. The entire signals were recorded using the Call–Purcell–Meiboom–Gill (CPMG) sequence with a spin–spin relaxation delay of 10 ms. All ^1^H NMR original spectra were corrected for zero, baseline and phase with Topspin (V3.0, Bruker Biospin, Berlin, Germany). Doublet calibration was carried out using a low field of α-glucose (δ 5.23). Peaks for water (5.17–4.47 ppm) and urea (6.60–5.42 ppm) were removed. The entire original spectra were processed into an ASC II document with MestReNova software (Mestrelab Research, Barcelona, Spain), and exported as a .txt file. All ^1^H NMR original spectra were processed using Chenomx NMR suite 7.5 (Chenomx Inc., Edmonton, Canada), using the Chenomx software library of compounds for automatic matching, and the displacement and peak shape of the compounds were recorded.

### 2.5. Multivariate statistical analysis

The normalized data (.txt file) were subjected to multivariate statistical analysis using R software (version 3.02) including principal component analysis and supervised orthogonal signal correction partial least-squares discriminant analysis (OSC-PLS-DA). A repeated 10-fold cross-validation and a permutation test (*n* = 200) were applied in the OSC-PLS-DA model. The quality of the model was evaluated by R2 and Q2, which indicate the total explained variation and represents the model predictability (Li et al. 2015).

### 2.6. Pathway analysis

Metabolic pathway analysis (MetPA) was performed by Metaboanalyst (http://www.metaboanalyst.ca) to indicate disturbed metabolism and construct network diagrams based on correlations observed between differentially regulated metabolites (Xia et al. 2009, 2012).

## 3. Results

### 3.1. Clinical and pathological information of experimental cows

At 60–90 days postpartum, plasma concentrations of NEFAs and total estrogens in the two groups differed significantly (*P* < 0.05, *P* < 0.01, respectively; Table 1). It shows that the lack of hormone in anestrous cows and the significant increase in NEFA are worth discussing. Within an elective waiting period of 60−90 days postpartum mean plasma concentration of total estrogens was significantly higher in estrus cows as compared to anestrus cows (71.2 ± 26.0 and 42.4 ± 16.7 pg/mL, respectively).

**Table 1.** Blood parameters of both groups of Holstein cows used (*n* = 10 per group).

Time point	Parameters	Estrus	Anestrus
14–21 days postpartum	Glucose (mmol/L)	3.25 ± 0.38	3.19 ± 0.24
	BHBA (mmol/L)	0.55 ± 0.13	0.65 ± 0.14
	NEFA (mmol/L)	0.32 ± 0.11	0.42 ± 0.10
60–90 days postpartum	Glucose (mmol/L)	3.43 ± 0.20	3.52 ± 0.33
	BHBA (mmol/L)	0.48 ± 0.15	0.48 ± 0.09
	NEFA (mmol/L)	0.22 ± 0.15*	0.40 ± 0.15
	E2 (pg/mL)	71.2 ± 26.0**	42.4 ± 16.7
	P4 (nmol/L)	5.4 ± 2.1	4.3 ± 1.7

Note: Keys: BHBA – beta-hydroxybutyric acid; NEFA – non-esterified fatty acids; E2 – total estrogens; P4 – progesterone. Values followed by (*) and (**) represent significant differences of *P* ≤ 0.01 and *P* ≤ 0.05, respectively.

### 3.2. Plasma metabolite profile

**Figure 1.**
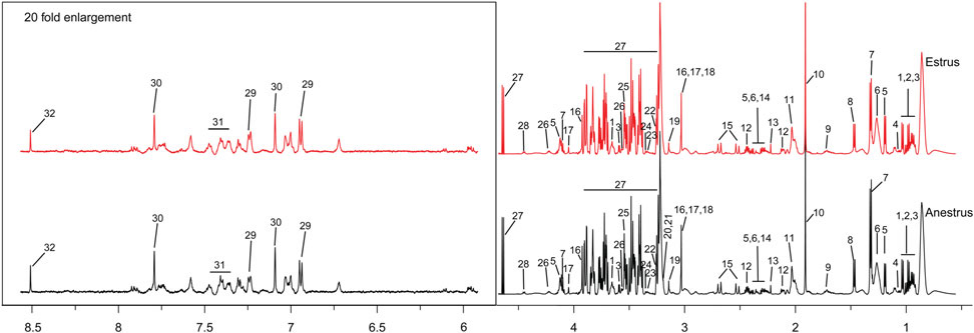
Typical 600 MHz ^1^H NMR spectra of plasma from estrus group and anestrus group. Metabolites: (1) Isoleucine; (2) Leucine; (3) Valine; (4) 3-Hydroxyisobutyrate; (5) 3-Hydroxybutyrate; (6) 3-Hydroxyisovalerate; (7) Lactate; (8) Alanine; (9) Arginine; (10) Acetate; (11) N-Acetylglycine; (12) Glutamine; (13) Levulinate; (14) Pyruvate; (15) Citrate; (16) Creatine; (17) Creatinine; (18) Creatine phosphate; (19) Dimethyl sulfone; (20) Choline; (21) Phosphatecholine; (22) Betaine; (23) Methanol; (24) Theophylline; (25) Glycine; (26) Threonine; (27) Glucose; (28) Lactose; (29) Tyramine; (30) Histidine; (31) Phenylalanine; (32) Formate.

**Figure 2.**
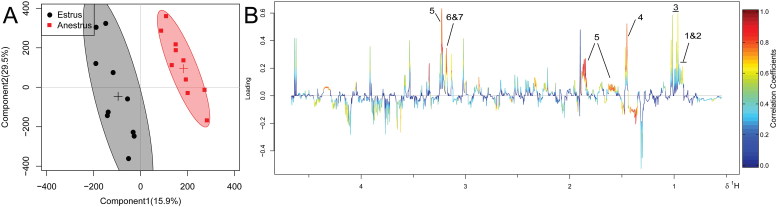
An OSC-PLS-DA score plot (A) and color-coded coefficient loadings plots (B) of metabolomic profiles. The circles in 2A represent the anestrus and estrous groups, respectively. In panel 2B, peaks with positive maxima represent metabolites with the largest correlation coefficients. Metabolites: (1 and 2) Isoleucine and Leucine; (3) Valine; (4) Alanine; (5) Arginine; (6 and 7) Choline and Phosphatecholine.

**Figure 3.**
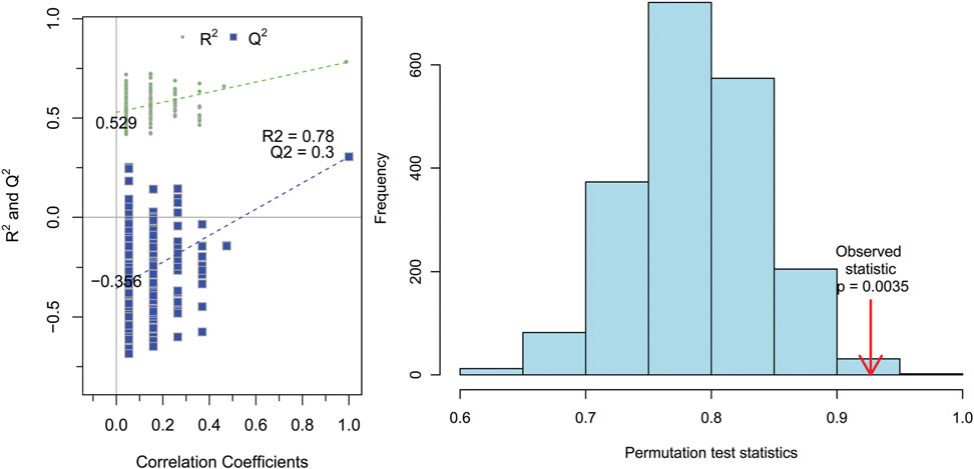
(A) OSC-PLS-DA scatter plots: R2 and Q2 values represent the interpretability and predictability of the model, respectively, where the closer the values are to 1, the more accurate the model. (B) Histograms for permutation test scores (*n* = 200).

Representative ^1^H NMR spectra of plasma of estrous and anestrous cows are shown in Figure 1. The OSC-PLS-DA scores plot (Figure 2(A)) illustrated a distinct separation between estrus and anestrus groups along the first principal component. The color-coded coefficient loadings plots (Figure 2(B)) revealed differential abundances in the following metabolites: isoleucine, leucine, valine, alanine, arginine, choline and phosphatecholine. Table 2 showed the identified metabolites. An OSC-PLS-DA model (Figure 3) was constructed from the plasma data from the two groups. The scatter plots of statistical validation were obtained (Figure 3(A); R2Y = 0.78 and Q2 = 0.3) and density plots of permutation test statistics (Figure 3(B)) indicate a successful OSC-PLS-DA model. There were significant differences revealed between the two groups by R2X, Q2, OSC-OPLS-DA and permutation tests.

**Table 2.** Assignment results of the identified metabolites in anestrus group (*n* = 10) compared to estrous group (*n* = 10).

No.	Metabolite	Assignments	Chemical shift (ppm)	Tendency
1	Isoleucine	δCH_3_, βCH_3_, γCH_2_	0.90 (d)	↑
2	Leucine	δCH3, δCH3, γCH, αCH_2_	0.95 (d)	↑
3	Valine	γCH_3_, γCH_3_, βCH, αCH	1.00 (m)	↑
4	Alanine	βCH_3_, αCH	1.48 (t)	↑
5	Arginine	γCH_2_, βCH_2_	1.55 (m), 1.80 (m), 3.20 (s)	↑
6	Choline	N(CH_3_)_3_, N–CH_2_	3.2 (d)	↑
7	Phosphatecholine	N(CH_3_)_3_	3.2 (s)	↑

Note: Multiplicity: *s* singlet, *d* doublet, *t* triplet, *m* multiplet. ‘↓’ indicates the content in group anestrus was lower than in group estrous.

### 3.3. Pathway analysis

**Figure 4.**
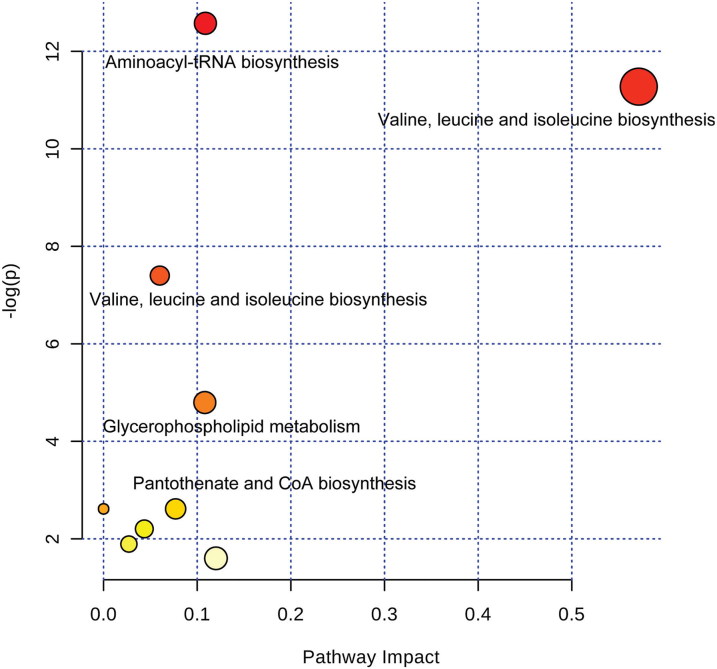
Bubble plots of the metabolic pathways affected in estrous vs. anestrous subjects. The lighter and smaller bubbles represent least affected pathways, whereas the larger and darker bubbles represent the more markedly affected pathways. A flowchart of the biosynthetic pathways is given for leucine, isoleucine and valine.

The biosynthetic pathways of the differentially abundant plasma metabolites between estrous and anestrous cows were analyzed using MetPA (Figure 4). The most markedly affected pathways between these two groups are those involved in valine, leucine and isoleucine biosynthesis.

## 4. Discussion

### 4.1. Amino acid metabolism

Leucine, isoleucine and valine are collectively referred to as the branched chain amino acids (BCAA). They have many important biological functions, including their role as precursors for protein synthesis (Gannon et al. 2018). BCAA can promote the production of IGF-1, while IGF-1 can act synergistically with LH and FSH to promote the expression of their receptors to promote follicular development (Butler and Butler 2001). In this study, the content of BCAA was decreased in anestrus cows. This finding does not exclude decreased IGF-1 production and associated obstructed follicle development.

Arginine catabolism plays a role as a precursor for nitric oxide (NO) synthesis via nitric oxide synthase (Gobert et al. 2000). NO plays an important role in the female reproductive system and in the release of Gonadotropin-releasing hormone (GnRH) (Knauf et al. 2001). GnRH may stimulate the pituitary gland to release follicle-stimulating hormone and luteinizing hormone, which together, can promote the growth and development of ovarian follicles. NO synthesis can be down-regulated as a consequence of increased arginine catabolism in the urea cycle, with consequences to GnRH release and the delay of estrus cycle. Therefore, decreased arginine concentration may be one of the reasons for the quiescence of dairy cows.

### 4.2. Glucose metabolism

Alanine can be converted into pyruvate within the alanine–glucose cycle, and pyruvate converted to glucose for body energy requirements via gluconeogenesis (Mccommis et al. 2015). In addition, pyruvate may also be converted to acetyl coenzyme A through oxidative decarboxylation and enter into the tricarboxylic acid cycle (TCA cycle). In this study, the concentrations of plasma glucose and alanine in anestrous cows were observed to be higher than in estrous cows. This suggests that glucose and alanine are not utilized to meet the higher energy demands during early lactation in anestrus cows. Xu C et al. (2016) also reported that the concentration of plasma alanine in the NEB anestrous cows was higher than estrous cows. This may be due to the reduced plasma glucose content in NEB cows, and as a result, the body promotes glucose production by increasing alanine content.

### 4.3. Lipid metabolism

Choline phosphorylation produces phosphatecholine (PC). Hydrolysis of PC produces choline and phosphatidic acid (PA). PA activates Raf-1 by direct interaction with Ras. Raf-1 leads to mitogen-activated protein kinase (MAPK) activation (Gallegoortega et al. 2009). The RAS-MAPK signal transduction pathway mainly promotes survival by stimulating the expression of anti-apoptotic proteins or genes. MAPKs in oocytes play an important role in microtubule organization, spindle formation, chromosome activity and maintenance of meiotic arrest in MII (Rodriguez-González et al. 2003). Therefore, the content of phosphocholine and phosphocholine in the blood of this study was reduced, lipid metabolism was affected, resulting in insufficient ovarian energy supply, and may affect the RAS-MAPK signal transduction pathway, thereby inhibiting follicular development, resulting in bovine ovarian quiescence.

In this study, anestrous cows were shown to have a higher concentration of plasma NEFAs relative to estrous cows, albeit below the upper limit of the reference range in both groups, suggesting a slightly higher rate of lipid mobilization. Of note, toxic effects of high NEFA concentrations on the oocyte have been shown earlier (Butler 2005) consistent with the high concentration of NEFAs in anestrus cows in this study.

## 5. Conclusion

In this study, 1H NMR spectrometry was used to identify differential metabolite abundances between estrus and anestrus EB dairy cows. The metabolites identified included relatively high plasma levels of BCAA, alanine, arginine, choline and PC in anestrus cow, implying differences in glucose, amino acid and lipid metabolic pathways. These different metabolites will provide a new direction for research into the causal mechanisms of ovarian quiescence in postpartum cows.
